# An innovative machine learning based on feed-forward artificial neural network and equilibrium optimization for predicting solar irradiance

**DOI:** 10.1038/s41598-024-52462-0

**Published:** 2024-01-25

**Authors:** Ting Xu, Mohammad Hosein Sabzalian, Ahmad Hammoud, Hamed Tahami, Ali Gholami, Sangkeum Lee

**Affiliations:** 1 School of Economics and Management, Hubei Engineering University, Hubei, 430000 China; 2 Department of Mechanical Engineering, Faculty of Engineering, University of Santiago of Chile (USACH), Avenida Libertador Bernardo O’Higgins 3363, 9170022 Santiago, Chile; 3 Department of Medical and Technical Information Technology, Bauman Moscow State Technical University, Moscow, Russia; 4 Department of Mathematics and Natural Sciences, Gulf University for Science and Technology, Mishref Campus, Kuwait; 5 School of Industrial and Information Engineering, Politecnico Di Milano, 20133 Milan, Italy; 6 Department of Electrical Engineering, Faculty of Technology and Engineering, Central Tehran Branch, Islamic Azad University, Tehran, Iran; 7 Department of Computer Engineering, Hanbat National University, Daejeon, 34158 South Korea

**Keywords:** Computational science, Computer science, Information technology

## Abstract

As is known, having a reliable analysis of energy sources is an important task toward sustainable development. Solar energy is one of the most advantageous types of renewable energy. Compared to fossil fuels, it is cleaner, freely available, and can be directly exploited for electricity. Therefore, this study is concerned with suggesting novel hybrid models for improving the forecast of Solar Irradiance (I_S_). First, a predictive model, namely Feed-Forward Artificial Neural Network (FFANN) forms the non-linear contribution between the I_S_ and dominant meteorological and temporal parameters (including humidity, temperature, pressure, cloud coverage, speed and direction of wind, month, day, and hour). Then, this framework is optimized using several metaheuristic algorithms to create hybrid models for predicting the I_S_. According to the accuracy assessments, metaheuristic algorithms attained satisfying training for the FFANN by using 80% of the data. Moreover, applying the trained models to the remaining 20% proved their high proficiency in forecasting the I_S_ in unseen environmental circumstances. A comparison among the optimizers revealed that Equilibrium Optimization (EO) could achieve a higher accuracy than Wind-Driven Optimization (WDO), Optics Inspired Optimization (OIO), and Social Spider Algorithm (SOSA). In another phase of this study, Principal Component Analysis (PCA) is applied to identify the most contributive meteorological and temporal factors. The PCA results can be used to optimize the problem dimension, as well as to suggest effective real-world measures for improving solar energy production. Lastly, the EO-based solution is yielded in the form of an explicit formula for a more convenient estimation of the I_S_.

## Introduction

### Background

Recent advances in computational and engineering domains have provided reliable responses to various problems in human modern life^1–5^. Intelligent tools, sophisticated simulation packages, and soft computing approaches are evident examples of these advances^6–9^. In the field of energy, engineers have successfully employed these tools and methodologies to improve the sustainable development of renewable energy systems^10–13^. Recently, solar energy has been introduced as an outstanding renewable source due to its numerous benefits like environmental friendliness, universality, high capacity, and inexhaustible supply^14,15^. Scholars attempted to evaluate the solar energy production pattern by forecasting related parameters such as I_S_. However, the evaluation of these factors required reliable approaches to handle non-linear calculations because of the existing many involved parameters^16,17^. Today, Machine Learning (ML) models proved to have an impressive approach to handling non-linear calculations^18–20^. In terms of forecasting tasks, ML methods can be used to conduct complicated mathematical relationships and provide exact solutions. Artificial Neural Network (ANN)^21,22^, Support Vector Machine (SVM)^23,24^, decision trees^25,26^, and neuro-fuzzy tools^27,28^ are among the most popular ML algorithms utilized for prediction aims related to solar energy calculations.

### Literature review

ML algorithms provided fast, inexpensive, and reliable solutions, which motivated experts to take advantage of them in the forecasting tasks^29–33^. Kim, Seong and Choi^34^ utilized ANN Models to forecast the energy consumption of an actual air handling unit and the appropriate result was obtained. Bhatt and Gandhi^35^ used two different statistical and ANN models to forecast the energy consumption in the wind power plants and the error of the feed-forward neural network was determined to be around 9.85%. Yin, Jia, Wu, Dai and Tang^36^ used a feedforward ANN model for forecasting tasks in the case of energy demand and the mean relative error value of the forecast was determined to be 1.58%. Malvoni, De Giorgi and Congedo^37^ utilized an SVM method to forecast the data from the Photovoltaic (PV) power. This method was also utilized in Ref. for forecasting wind energy production in Estonia and compared to other traditional methods like Behavior-Driven Development (BDD), appropriate results were obtained.

Optimization-oriented efforts form lots of studies in the engineering literature^38–40^. In particular, many scholars have suggested metaheuristic algorithms for optimization purposes^41,42^. They can also serve to optimize traditional ML methods like ANN and ANFIS^43^. These algorithms were concerned in the case of renewable energy analysis in many studies previously^44,45^ such as solar power energy^46^ and wind energy^47,48^. Computational problems such as local minima can be removed by using the metaheuristic-based hybrids^29^. In the following, previous works related to the use of metaheuristic algorithms to optimize ML methods in the case of energy forecasting tasks are briefly summarized. Moayedi and Mosavi^49^ utilized an innovative metaheuristic approach (Electromagnetic Field Optimization (EFO)) to optimize a neural network and proved that the EFO-supervised neural network algorithm can appropriately mine a dataset of nonlinearly tuning the network elements. Abedinia, Amjady and Ghadimi^50^ utilized a productive engine consisting of a metaheuristic optimizer, namely shark smell optimization to optimize ANN. They claimed that this hybrid method had better performance compared to other conventional predictors such as ANN with lower normalized Root Mean Square Errors (RMSEs) by about 27% compared to ANN and other traditional methods. Galván, Valls, Cervantes and Aler^51^ used a multi-objective Particle Swarm Optimization (PSO) method to enhance the ANN method and observed that the PSO optimizer had outstanding results compared to traditional ANN. Tran, Luong and Chou^52^ introduced a new model namely Evolutionary Neural Machine Inference Model (ENMIM) consisting of different models of Least Squares Support Vector Regression (LSSVR), and the Radial Basis Function Neural Network (RBFNN) together with Symbiotic Organism Search (SOS) for obtaining optimized tuning parameters. This approach was proved to be a promising alternative for the energy management tasks. Halabi, Mekhilef and Hossain^53^ demonstrated that the algorithm introduced in^54^ can be coupled with an ANFIS system in the case of I_S_ predictions. Louzazni, Khouya, Amechnoue, Gandelli, Mussetta and Crăciunescu^55^ have proven the competency of the algorithm of firefly to evaluate the solar energy harvesting parameters and observed that the firefly algorithm was very reliable in the case of solar energy forecasting. Bechouat, Younsi, Sedraoui, Soufi, Yousfi, Tabet and Touafek^56^ also concerned the PSO and Genetic Algorithm (GA) in this case. Zhou, Moayedi and Foong^57^ have studied the limitation of neural computing approaches, for example, local minima, and suggested a novel metaheuristic method namely Teaching–Learning-Based Optimization (TLBO) for enhancing a Multi-Layer Perceptron Neural Network (MLPNN). They observed that, by using TLBO method, the prediction error is reduced by 19.89% compared to the ANN approach. Vaisakh and Jayabarathi^58^ utilized a hybrid approach called the deer hunting optimization algorithm as well as grey wolf optimization to adjust the structure of ANNs, which was used for solar energy calculations. Their achievements reflected a notable improvement attained by the tested optimizer. Abedinia, Amjady and Ghadimi^59^ have used a neural network algorithm enhanced by a metaheuristic algorithm as the hybrid method for the forecasting tasks in the case of solar energy harvesting, and appropriate results were obtained. Abdalla, Rezk and Ahmed^60^ have successfully utilized Wind-Driven Optimization (WDO) to track the elements of photovoltaic systems and justified that the mentioned algorithm had better results compared to many traditional optimization techniques such as cuckoo search.

### Motivation, novelty, and objective

The above literature shows the necessity of utilizing modern tools and techniques for coping with intricate engineering problems^61–65^. In this sense, different ML models have great contributions to the concept of renewable energy, particularly for SE-related predictions. On the other hand, metaheuristic algorithms have been recommended for optimal development of ML models. Based on the previous literature, incorporating metaheuristic optimizers with ML models such as ANN helps to avoid computational drawbacks, and therefore, is becoming a research hotspot in this way^66^. However, a gap of knowledge emerges when these studies mostly focus on earlier metaheuristic methods such as PSO and GA^67,68^, because the metaheuristic family is being extended by new potential members. This gap calls for evaluating the capability of newer hybrid models to improve SE-related predictions. Hence, in this research, a novel potential metaheuristic technique named EO is employed through an FFANN framework to analyze the meteorological and temporal conditions and predict the I_S_. The EO algorithm here is responsible for best-tuning the FFANN’s weights (and biases) which connect the I_S_ to the environmental conditions. Moreover, to comparatively validate the performance of the EO, this algorithm is evaluated versus three benchmark optimizers including WDO, Optics Inspired Optimization (OIO), and Social Spider Algorithm (SOSA), as well as three algorithms of the EFO, Shuffled Complex Evolution (SCE), and Shuffled Frog Leaping Algorithm (SFLA) used in an earlier study by Moayedi and Mosavi^49^. Accuracy assessment is carried out using different criteria to rank them and distinguish the most competent model. Since the used models have not been applied to this problem before, the findings of this study can assist solar energy experts in the appropriate selection of predictive models. For more convenience, a mathematical formula is also extracted from the EO-FFANN model to eliminate the need for computer-aided implementations in predicting the I_S_. Moreover, a well-known statistical technique called Principal Component Analysis (PCA) is applied to identify the most contributive meteorological and temporal parameters, and therefore, to optimize the dimension of the problem.

To sum up, the main strengths and novelties of this study can be highlighted as follows:

- Evaluating the applicability of ensemble learning theory for predicting the I_S_ as a crucial parameter of renewable (solar) energy,
- Employing the EO metaheuristic algorithm to create a novel FFANN-based model whose absence is considered a gap of knowledge in the literature on I_S_ prediction,
- Introducing the optimal configurations (i.e., population size and No. of iterations) for the used models,
- Exposing various environmental and temporal conditions as key parameters in the I_S_ prediction and determining the principal dataset components using the PCA method which has not been performed in the previous literature. In addition to optimizing the problem dimension, the results of the PCA can be regarded for suggesting real-world measures (attributing to the key parameters) to maximize solar energy production.
- Conducting a comparative assessment by evaluating six other metaheuristic algorithms in this study (i.e., WDO, OIO, and SOSA) and previous literature (i.e., EFO, SCE, and SFLA). It makes this study a suitable benchmark for future applications of hybrid models and appropriate model selection by energy experts,
- Developing a monolithic explicit formula from the proposed EO-FFANN model to be used as a convenient method for predicting the I_S_.

Overall, the achievements of this research can greatly contribute to the body of knowledge (from both data and methodology perspectives) that deals with solar energy modeling. Performing several optimization ideas carried out in this study can be helpful to reduce the complexities (i.e., computational costs) in the way of proper I_S_ prediction.

In the following, the study continues by introducing the used materials and methods in Sect. 2, presenting the results and discussion in Sect. 3, followed by providing conclusions in Sect. 4.

## Materials and methods

### Dataset and splitting

**Figure 1:**
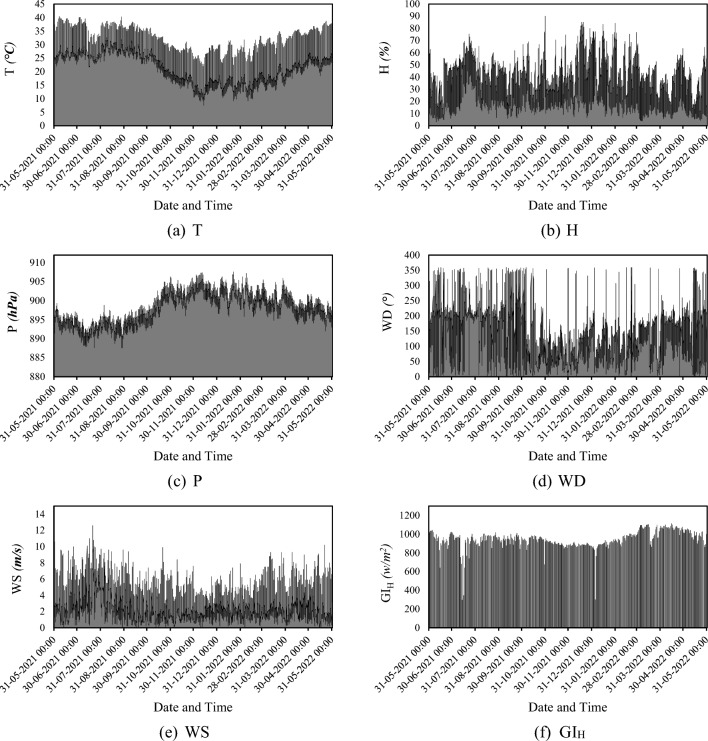
Time-series of the GI_H_ and meteorological parameters.

From previous studies, it is evident that the amount of received I_S_ is a function of various meteorological conditions^69,70^. In this work, this amount is represented by a so-called parameter Global Horizontal Irradiance (GI_H_) which is measured for Yemen. Along with the GI_H_, the records of five meteorological factors, namely: Air Temperature (T), Relative Humidity (H), Surface Pressure (P), Wind Direction (WD), and Wind Speed (WS) are downloaded from the Solcast community (https://solcast.com/). All measurements are hourly within one year (2021-05-31 to 2022-06-01). Figure 1 shows the time series of the T, H, P, WD, WS, and GI_H_.

In addition to these five parameters, three temporal inputs, namely Month (m), Day (d), and Hour (h) are also considered influential parameters. When this dataset is exposed to the considered ML models, the influential parameters (i.e., m, d, h, T, H, P, WD, and WS) play the role of inputs, while the GI_H_ is the target of the system. Therefore, the used models explore the relationship between the temporal and meteorological parameters to understand and predict the hourly GI_H_. Table 1 gives the results of the statistical analysis performed on the used dataset.

**Table 1:** Correlation factors showing the proportionality of the parameters.

	T (*°C*)	H (*%*)	P (*hPa*)	WD (*°*)	WS (*m/s*)	GI_H_ (*w/m*^*2*^)
Mean	25.64	30.64	898.12	132.60	3.38	273.85
Standard error	0.07	0.18	0.04	0.86	0.02	3.80
Median	26.10	28.10	898.30	121.00	3.00	10.00
Mode	28.30	16.30	901.20	56.00	1.90	0.00
Standard deviation	6.78	16.49	3.99	80.83	1.98	356.42
Sample variance	45.94	271.87	15.93	6532.69	3.92	127,036.86
Kurtosis	−0.69	−0.20	−0.88	−0.24	0.50	−0.84
Skewness	−0.15	0.63	−0.10	0.60	0.85	0.88
Range	33.00	87.00	20.00	360.00	12.60	1113.00
Minimum	7.50	3.10	887.60	0.00	0.00	0.00
Maximum	40.50	90.10	907.60	360.00	12.60	1113.00
Sum	225,706.00	269,723.30	7,906,112.80	1,167,256.00	29,760.90	2,410,715.00
Count	8803.00	8803.00	8803.00	8803.00	8803.00	8803.00

As per Table 1, a total of 8803 records exist in the dataset. These records are split into two sub-sets for creating the training and testing sets. The training set is required to provide the training material for the models, and the testing set examines the generalizability of the models. Based on previous works, 80:20 ratio is applied to split the dataset, meaning that 7042 records exist in the training set, and 1761 records exist in the testing set.

### Applied algorithms

#### Overview of EO

As the name implies, the EO is an optimization technique that mimics specific laws of physics to obtain an optimum solution^71^. It is a capable metaheuristic algorithm for dealing with problems with different levels of complexity. The search units of the EO are called particles each of which receives an initial concentration value as in Eq. (1):

1 $${C}_{j}= LB + r\times (UB- LB),$$

where $r$ is a random value in [0, 1]. Moreover, $LB$ and $UB$ are the lower and upper bounds of the space.

Similar to other population-based optimizers, the quality of the particles is reflected by a fitness value. They are then sorted, and the algorithm hires four of them which are distinguished by the highest fitness value. A fifth particle is also considered that represents the mean of these four particles.

The exponential term (*F*) of the algorithm is defined by Eqs. (2), (3), (4).

2 $$F = {CP}_{1} sign\left(r-0.5\right)({e}^{-\beta t}-1),$$

3 $$t= {(1- {R}_{iter})}^{({CP}_{2} \times {R}_{iter})},$$

4 $${R}_{iter}=\frac{iter}{T},$$

in which $\beta$ stands for the turnover rate,${CP}_{1}$ and ${CP}_{2}$ are controlling parameters for the exploration and exploitation phases, respectively.

Assuming ${G}_{CP}$ and $GP$ as a controlling parameter and the generation probability, respectively, generation rate is calculated by Eqs. (5) and (6).

5 $${R}_{G} = {G}_{0} {G}_{CP} \left({C}_{eq}- \beta C\right) F,$$

6 $${G}_{CP}= \left\{\begin{array}{c}0.5 {r}_{1} \,\,\,\,\,\,\,\,{r}_{2}\ge GP \\ 0 \,\,\,\,\,\,\,\,\,\,\,\,\,\,\,\,\,\,\,otherwise\end{array}\right.,$$

where ${C}_{eq}$ is the equilibrium pool, and ${r}_{1}$ and ${r}_{2}$ are random numbers in [0, 1].

Based on the above calculations, the solution is updated as in Eq. (7)^72^:

7 $${C}_{j} = {C}_{eq}+\left({C}_{j}-{C}_{eq}\right) \times F+\left(1- F\right)\frac{{R}_{G}}{\beta V},$$

where $V$ is the considered unit.

#### Comparative algorithms

Wind-driven optimization was first introduced by Bayraktar, Komurcu and Werner^73^ in 2010 for electromagnetics applications. The WDO relies on the air parcel's movement in hyper-dimensional space. These movements are supposed to be affected by four natural forces of Coriolis force, gravitational force, frictional force, and pressure gradient force. Also, by taking into consideration the ideal gas equation, the position (as well as the velocity) of the air parcels is updated to find the best responses. Scholars like Moayedi, Bui and Ngo^74^ and Bayraktar^75^ have successfully used the WDO for optimizing the neural parameters.

As a physic-based scheme, the OIO was suggested by Kashan^76^ in 2014. It is inspired by optics (a law in physics) which works by a group of artificial light-related stuff. After randomly generating the fixed number of individuals, the initial position of the light points is determined. Each point is then put in front of an artificial mirror and its image is created in the search space with a certain distance from the main axis. The position of the image is then updated to be a new solution This process continues until a stopping criterion is satisfied^77^.

The SOSA, as the name implies, takes the idea from the food-seeking action of social spider, introduced by James and Li^78^ in 2015. In this method, the solution space is considered a hyper-dimensional spider web that the agents (i.e., spiders) can move on it. As assumptions, the agents have regular interaction with each other and every position in this area corresponds to a possible solution^79^. Each spider distinguishes itself by the position and fitness value. The agents possess a memory to hold three basic attributes: all possible vibration intensities are positive, (ii) the larger the fitness values mean more intense vibrations, and (iii) once the best solution is getting close, the vibration does not experience excessive increase.

Mathematical details pertaining to the above algorithms can be found in the literature (like the WDO^60,80^, OIO^81,82^, and SOSA^83,84^).

### Evaluation method

Statistical indices are normally used for evaluating the accuracy of ML models. In this work, RMSE along with Mean Absolute Error (MAE) is used to indicate the prediction error. Given ${GI}_{H {i}_{real}}$ and ${GI}_{H {i}_{predict}}$ as the real and predicted GI_H_s, respectively, Eqs. (8) and (9) formulate the RMSE and MAE as follows:

8 $$RMSE=\sqrt{\frac{1}{S}\sum_{i=1}^{S}{({GI}_{{Hi}_{real} }-{GI}_{{Hi}_{predict} })}^{2}},$$

9 $$MAE=\frac{1}{S}\sum_{i=1}^{S}\left|{GI}_{{Hi}_{real} }-{GI}_{{Hi}_{predict} }\right|,$$

where S stands for the size of the set.

Moreover, a so-called correlation indicator “Pearson Correlation Coefficient (R)” is designated as per Eq. (10) to reflect the agreement between reality and prediction.

10 $$R=\frac{\sum_{i=1}^{S}({GI}_{{Hi}_{predict} }-\overline{{GI }_{{H}_{predict} }})({GI}_{{Hi}_{real} }-\overline{{GI }_{{H}_{real} }})}{\sqrt{\sum_{i=1}^{S}{({GI}_{{Hi}_{predict} }-\overline{{GI }_{{H}_{predict} }})}^{2}}\sqrt{\sum_{i=1}^{S}{({GI}_{{Hi}_{real} }-\overline{{GI }_{{H}_{real} }})}^{2}}}\times 100,$$

## Results and discussion

### Hybridization of algorithms

When the FFANN is hybridized with a metaheuristic algorithm, the basic idea is to optimize its weights and biases to establish the best relationship between the target and input parameters. In this research, the FFANN is optimized by the EO algorithm, as well as OIO, WDO, and SOSA. The metaheuristic algorithms are able to find the solution in an iterative process.

**Figure 2:**
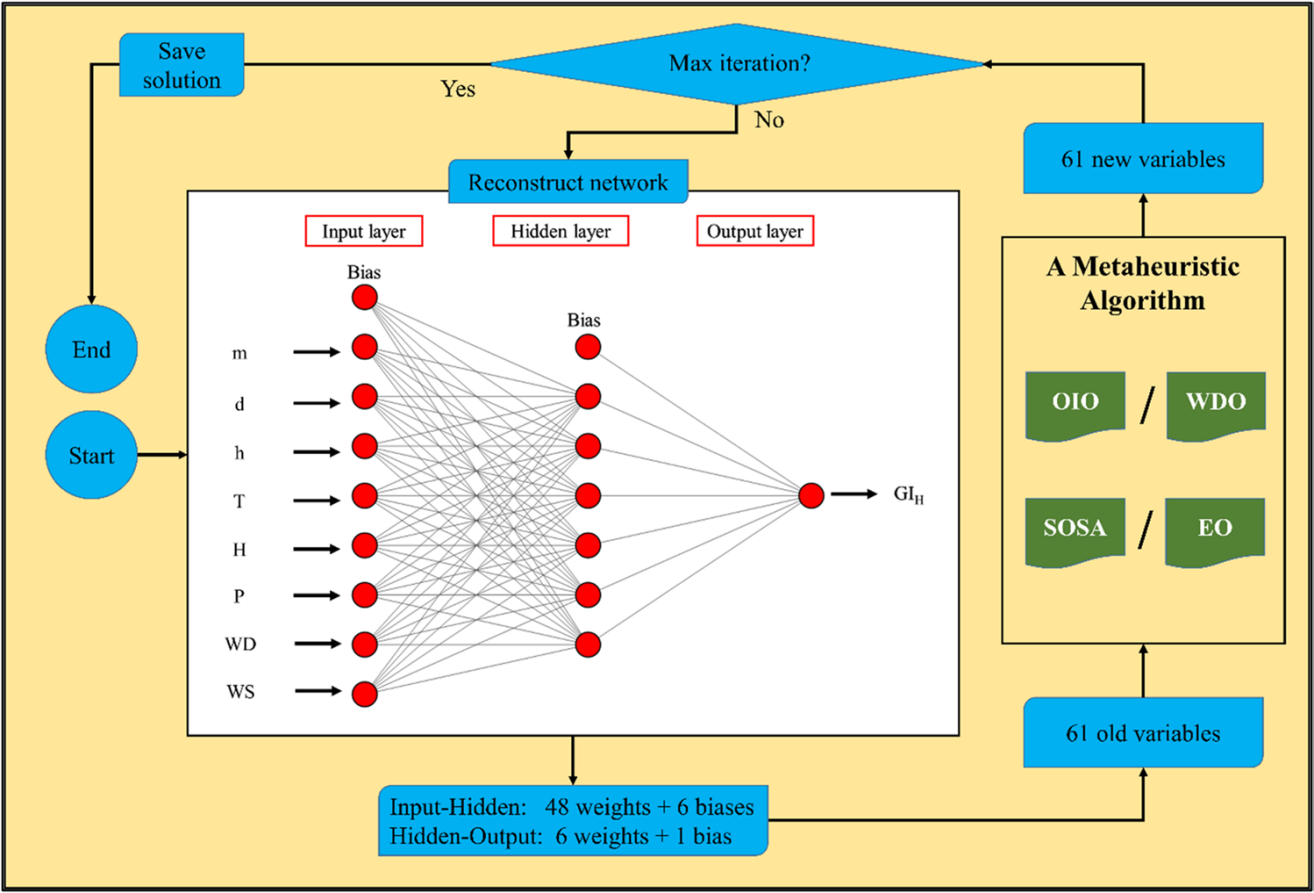
Optimization process of the hybrid models.

The used FFANN is represented by an MLPNN (8,6,1) model indicating a three-layered neural network with 8 input neurons in the first layer, 6 hidden neurons in the middle layer, and 1 output neuron in the last layer. The activation functions in the middle and last layers are Tansig and Purelin, respectively. This configuration is obtained after an extensive trial-and-error effort. A topology of the used FFANN is embedded in Fig. 2. According to this figure, this network has a total of 61 weights and biases which are optimized by the metaheuristic algorithm. In this process, the training dataset is used solely. First, the mathematical equation of the FFANN is extracted and is considered as the problem function. Next, a metaheuristic algorithm is run to tune the FFANN equation (i.e., weights and biases) so that the training RMSE is minimized by 1000 iterations. In each iteration, new 61 variables construct the FFANN, and the training RMSE is calculated. Note that, each of the EO, OIO, WDO, and SOSA algorithms were implemented with different population sizes (varying from 50 to 700) and it was observed that the best population size for them is 400, 200, 100, and 200, respectively.

## Optimization results

**Figure 3:**
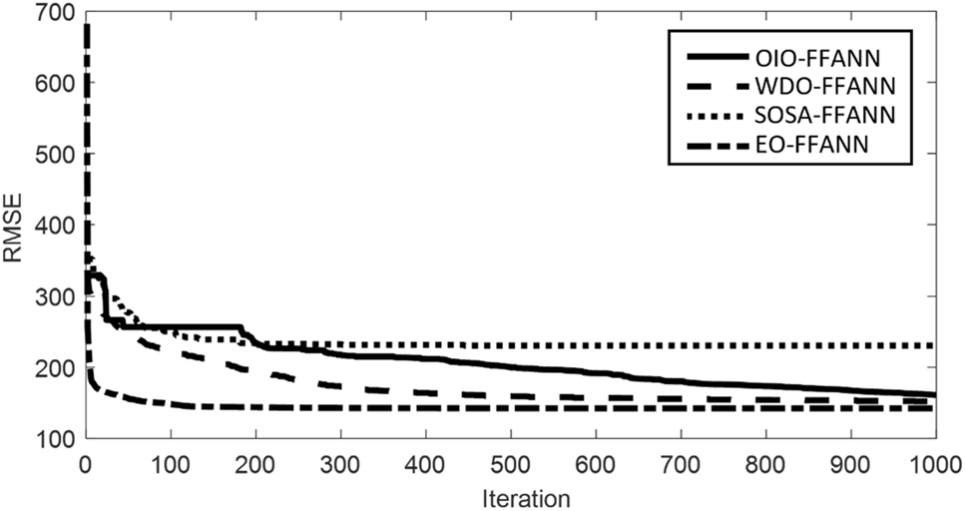
Optimization curves of the OIO-FFANN, WDO-FFANN, SOSA-FFANN, and EO-FFANN algorithms.

Figure 3 shows the optimization process for the used algorithms (with the mentioned best population sizes) that are iterated 1000 times. From the comparison of the curves, it is immediate that the EO has reached a higher quality of solution due to the minimum RMSE error. While the solutions of the WDO and OIO are very close, the SOSA has found the solution with considerably higher error. Note that this process was carried out using the training set only because the testing set should be kept away from the models in this stage. In the next two sections, the training and testing results are assessed using the accuracy methods.

### Training accuracy

**Figure 4:**
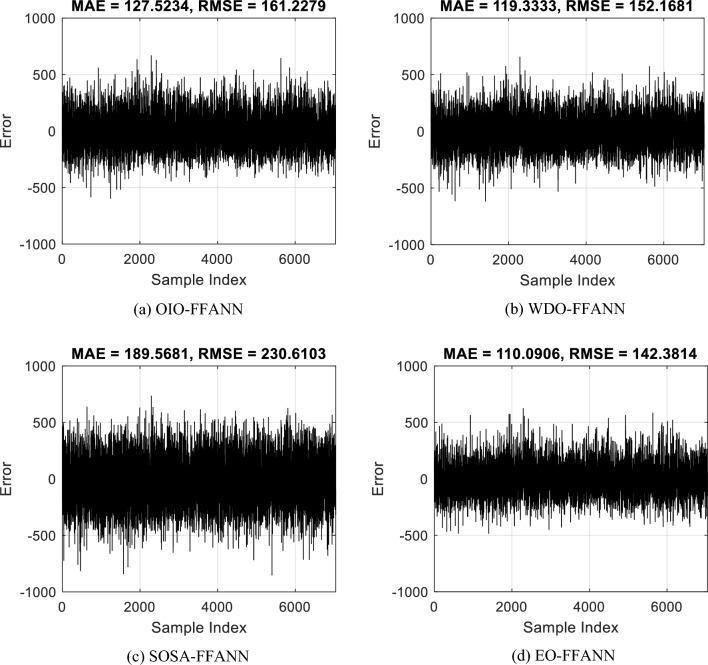
Training errors of (**a**) OIO-FFANN, (**b**) WDO-FFANN, (**c**) SOSA-FFANN, and (**d**) EO-FFANN.

Figure 4 forms part of the training results as the final RMSEs are the training RMSEs. Having the order of algorithms as OIO-FFANN, WDO-FFANN, SOSA-FFANN, and EO-FFANN, training RMSEs were 161.22, 152.16, 230.61, and 142.38 *w/m*^*2*^.

Figure 4 illustrates the error values for the 7042 records in the training set. Each single value in this figure indicates the pure difference between ${GI}_{H {i}_{real}}$ and ${GI}_{H {i}_{predict}}$. Hence, the lower the value, the higher the accuracy. In a glance, it can be seen that the results of the EO are better positioned around the Y = 0 line. Quantitatively speaking, the training MAEs were 127.52, 119.33, 189.56, and 110.09 *w/m*^*2*^.

The calculated values of the RMSE and MAE indicated an acceptable level of error for all used models. As for the R index, the values were 0.89, 0.90, 0.76, and 0.91 which demonstrate a significant level of agreement between the reality and prediction results of all four models. However, again, the superiority of the EO algorithm is obvious in terms of the R, too. It was the only model that achieved a correlation larger than 90%.

### Testing accuracy

This section shows the performance of the OIO-FFANN, WDO-FFANN, SOSA-FFANN, and EO-FFANN when they are subjected to the 1761 records in the testing set. This process demonstrates the power of the trained models in dealing with unseen environmental conditions for estimating hourly GI_H_.

**Figure 5:**
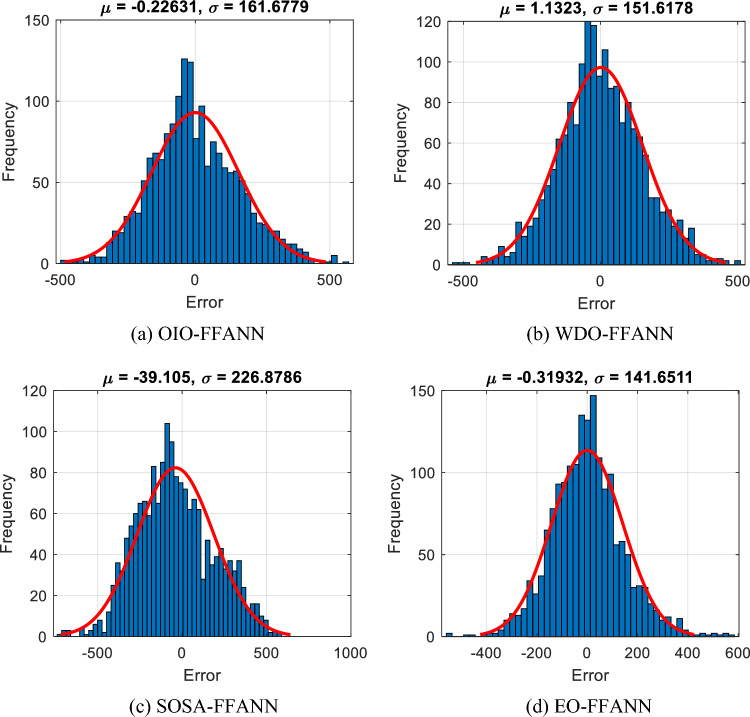
Testing histogram of errors of (**a**) OIO-FFANN, (**b**) WDO-FFANN, (**c**) SOSA-FFANN, and (**d**) EO-FFANN.

From the obtained RMSEs of 161.63, 151.57, 230.16, and 141.61 *w/m*^*2*^, it is quantitatively inferred that the testing results enjoy a satisfying level of accuracy. Figure 5 illustrates the statistics of the testing errors. In these histogram charts, the higher the frequency of 0 error, the better the accuracy. As is seen, the distribution is almost normal for all models. It professes the high quality of testing results. Besides, the MAEs of 127.52, 118.72, 188.20, and 108.07 *w/m*^*2*^ indicate a low level of average errors.

**Figure 6:**
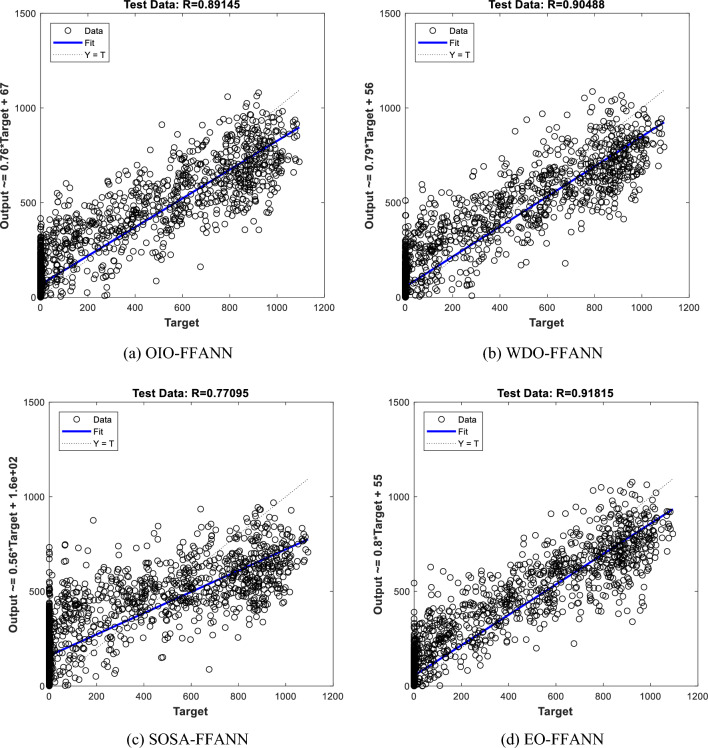
Correlation diagrams for testing set of (**a**) OIO-FFANN, (**b**) WDO-FFANN, (**c**) SOSA-FFANN, and (**d**) EO-FFANN.

Figure 6 shows the correlation diagrams of the testing set. The values on the horizontal and vertical axis represent the ${GI}_{H {i}_{real}}$ and ${GI}_{H {i}_{predict}}$, respectively. Hereupon, the ideal prediction happens when all points are positioned on the line x = y, and the R-value is 1. As per Fig. 6, all four models have performed a nice prediction and their calculated Rs were 0.89, 0.90, 0.77, and 0.91. Similar to the training stage, EO-FFANN is the only algorithm with a correlation above 90%.

### Accuracy comparison

**Figure 7:**
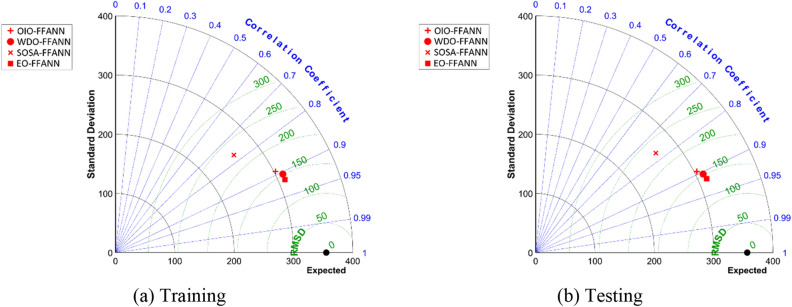
Taylor diagrams for (**a**) training and (**b**) testing sets.

It was in general shown that the EO-based model outperformed the benchmarks in both training and testing stages. In this section, the models are subjected to a more detailed comparison to rank them. For this purpose, Taylor diagrams are generated and presented in Fig. 7. These figures can simultaneously show the correlation (Correlation Coefficient) and error (RMSD = RMSE). As is seen, in both training and testing sets, the same pattern is obtained, and it means there is no discrepancy between the training and testing qualities. The EO-FFANN is distinguished by the lowest error and highest correlation, followed by WDO-FFANN and OIO-FFANN. As for SOSA-FFANN, this model has a considerable weakness in its performance in comparison with three other models. As per Fig. 7, the point of the SOSA-FFANN is separated from the others.

### PCA importance analysis

**Figure 8:**
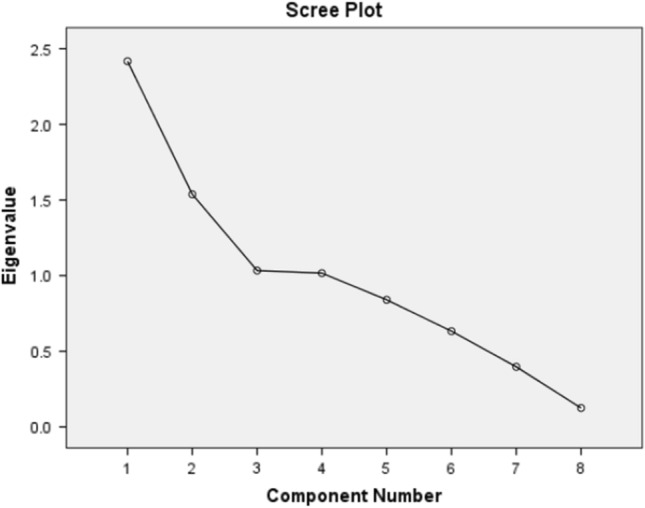
Scree plot of the PCA analysis.

In this section, an importance assessment is applied to the used dataset. The results of such efforts can be of great importance for the proper selection of input factors from the statistical point of view. The PCA technique^85^ is used to determine the most contributive factors for the GI_H_ prediction. Figure 8 shows the obtained scree plot, according to which, four components have an eigenvalue larger than 1. These four components are considered as principal components, and based on Table 2, cumulatively account for about 75% of the variance in the dataset.

**Table 2:** Total variance explained.

Component	Initial eigenvalues			Extraction sums of squared loadings			Rotation sums of squared loadings		
	Total	% of Variance	Cumulative %	Total	% of Variance	Cumulative %	Total	% of Variance	Cumulative %
1	2.417	30.213	30.213	2.417	30.213	30.213	2.141	26.764	26.764
2	1.537	19.218	49.431	1.537	19.218	49.431	1.796	22.451	49.215
3	1.034	12.919	62.350	1.034	12.919	62.350	1.040	12.999	62.214
4	1.016	12.706	75.056	1.016	12.706	75.056	1.027	12.842	75.056
5	0.840	10.501	85.557						
6	0.633	7.910	93.466						
7	0.397	4.967	98.434						
8	0.125	1.566	100.000						

Table 3 shows the results of the Varimax rotation method. In each of the four components, the factors with loading above + 0.70 and below −0.70 are selected. As is seen, Component 1 reflects T and H, Component 2 reflects P and WD, Component 3 reflects d, and Component 4 reflects m. Hence, it can be deduced that h and WS can be discarded for optimizing the dataset.

**Table 3:** Rotated component matrix from Varimax method.

	Component			
	1	2	3	4
m	−0.039	0.019	0.030	**0.983**
d	0.031	−0.012	**0.936**	0.032
h	0.655	−0.138	0.126	−0.039
T	**0.720**	0.565	−0.110	0.162
H	−*0.736*	0.094	0.316	−0.042
P	−0.301	−*0.868*	−0.010	−0.093
WD	−0.346	**0.794**	0.012	−0.099
WS	0.663	0.255	0.189	−0.113

Significant values bold and italics.

### A monolithic formula

In order to eliminate the need for implementing computer-based programs, this section provides a mathematical expression that is derived from the proposed model i.e., EO-FFANN, for predicting the GI_H_. The reason for considering EO-FFANN is that this model achieved the highest accuracy in the previous assessments. The formula is a monolithic relationship; however, it has two steps and the GI_H_ needs to be calculated in the second step.

Referring to the FFANN topology in Fig. 2, this equation is constructed from 61 weights and biases. The general inputs of this equation are m, d, h, T, H, P, WD, and WS that feed Eq. (11). With the help of Table 4, the outcomes of this equation are ${N}_{i}$ (i = 1, 2, …, 6) that feed Eq. (12) for calculating the GI_H_. In other words, Eq. (11) and Table 4 together express the process between the input and hidden layers of the FFANN, while Eq. (12) expresses the process between the hidden and output layers (see Fig. 2).

11 $${N}_{i}=Tansig({W}_{i1} \times m + {W}_{i2} \times d + {W}_{i3} \times h + {W}_{i4} \times T + {W}_{i5} \times H + {W}_{i6} \times P + {W}_{i7} \times WD + {W}_{i8} \times WS+ {b}_{i}),$$

12 $$\begin{aligned} GI_{H} = & \, 0.4187 \, \times \, N_{1} + \, 0.5094 \, \times \, N_{2} - \, 0.4479 \, \times \, N_{3} + \, 0.3594 \, \times \, N_{4} \\ & + \, 0.3102 \, \times \, N_{5} - \, 0.6748 \, \times \, N_{6} - \, 0.7620, \\ \end{aligned}$$

**Table 4:** Optimized internal parameters of the FFANN.

i	W_i1_	W_i2_	W_i3_	W_i4_	W_i5_	W_i6_	W_i7_	W_i8_	b_i_
1	0.6970	−0.4906	1.0125	0.6472	0.3959	0.4563	0.4315	0.5881	−1.7514
2	0.8025	0.0927	−0.0289	0.9087	0.5097	−0.9258	−0.3617	0.5838	−1.0509
3	−0.6994	0.8579	0.5630	0.2920	0.4559	−0.4183	0.8442	−0.5871	0.3503
4	0.6707	0.7543	−0.5810	−0.7533	−0.1749	−0.7363	−0.7554	−0.0166	0.3503
5	0.3433	−0.8881	−0.2029	0.9056	0.4033	−1.0450	−0.1589	−0.1411	1.0509
6	−0.7074	0.8272	0.7308	0.7629	−0.5780	0.5686	−0.2082	0.2572	−1.7514

### Strength, limitations, and future guidelines

This study presented novel applications of metaheuristic-empowered ML models for predicting I_S_. A valid dataset with various meteorological and temporal factors was applied for this purpose. The models were optimized in terms of their hyper-parameters such as the FFANN topology and population size of the metaheuristic algorithms. Therefore, it can be claimed that the used models are among the most optimum ones. The desirable level of accuracy obtained in this study proved the applicability of the applied models, however, a comparison showed that the EO-FFANN shows greater promise. This model achieved improvement when it is compared to previous studies. For instance, Moayedi and Mosavi^49^ used the EFO algorithm, along with the SCE and SFLA, to optimize a similar FFANN. These models reached an R-value (non-percentage) of 0.82132, 0.78046, and 0.75212, respectively, which are lower than the R values of the EO-FFANN in this work.

Presenting a simplified formula is another outcome of this study which enables the users to predict the *GI*_*H*_ without the need for computer-aided facilities. Furthermore, regarding the performed trial and error efforts in different stages, it can be said that this solution is captured carefully among numerous candidates.

In Sect. 3.6, the PCA model was applied to the dataset and its results highlighted the T, H, P, WD, d, and m as the most contributive input factors. As is known, reducing the dataset inputs from 8 to 6 results in lightening the computational burden due to the reduction in the problem dimension^86^. Considering this idea is highly recommended for future efforts towards improving the solution for the GI_H_ prediction.

However, this study encountered some limitations, too. About the used dataset, it includes the records from 2021-05-31 to 2022-06-01. Hence, updating this dataset with the most recent data (e.g., late 2022 and early 2023) could be of great interest to future efforts. It may help in enhancing the generalizability of the suggested models for new climate conditions. As far as the models are concerned, although the applied metaheuristic algorithms are among the recent members of this family, more algorithms have been developed lately. Comparing the results of the EO with the most recent metaheuristic algorithms would greatly help in updating the solutions, and probably, increasing the accuracy of GI_H_ prediction.

## Conclusions

The importance of analyzing environmental conditions is evident in the forecast of renewable energy potentials. This work was dedicated to optimizing solar energy simulation using state-of-the-art ML and feature selection strategies. An FFANN was optimally trained using different metaheuristic algorithms for predicting solar irradiance from meteorological and temporal parameters (including humidity, temperature, pressure, cloud coverage, speed and direction of wind, month, day, and hour). Assessing the prediction results revealed that the EO performs more accurately than the three optimization algorithms evaluated in this study (OIO, WDO, and SOSA), as well as three optimization algorithms (EFO, SCE, and SFLA) from the earlier literature. Therefore, the mathematical representation of the EO-FFANN was presented in the form of a predictive formula to be reliably used for practical GI_H_ predictions. Moreover, the PCA method could successfully analyze the datasets and address the T, H, P, WD, d, and m as the input factors that are most essential in forecasting solar irradiance. This part of the results can be regarded in the real world for enhancing the generation of solar energy. In conclusion, the findings of this study professed the efficiency of the PCA and metaheuristic techniques for optimizing the performance of ML models. However, some ideas were presented for future work toward coping with the limitations of the study, most notably updating the used dataset and predictive models.

## Data Availability

All data analysed during this study can be downloaded from the Solcast community (https://solcast.com/). Also, the used codes will be made available upon reasonable request from the authors.
